# Pathological Involvement of Protein Phase Separation and Aggregation in Neurodegenerative Diseases

**DOI:** 10.3390/ijms251810187

**Published:** 2024-09-23

**Authors:** Yinuo Wu, Biao Ma, Chang Liu, Dangdang Li, Guangchao Sui

**Affiliations:** 1 Aulin College, Northeast Forestry University, Harbin 150040, China; yinuowu@nefu.edu.cn; 2 College of Life Science, Northeast Forestry University, Harbin 150040, China; 2022112834bma@nefu.edu.cn (B.M.); lc1677315800@nefu.edu.cn (C.L.)

**Keywords:** neurodegenerative diseases, phase separation and aggregation, pathology, treatment

## Abstract

Neurodegenerative diseases are the leading cause of human disability and immensely reduce patients’ life span and quality. The diseases are characterized by the functional loss of neuronal cells and share several common pathogenic mechanisms involving the malfunction, structural distortion, or aggregation of multiple key regulatory proteins. Cellular phase separation is the formation of biomolecular condensates that regulate numerous biological processes, including neuronal development and synaptic signaling transduction. Aberrant phase separation may cause protein aggregation that is a general phenomenon in the neuronal cells of patients suffering neurodegenerative diseases. In this review, we summarize the pathological causes of common neurodegenerative diseases, including Alzheimer’s disease, Parkinson’s disease, and Huntington’s disease, among others. We discuss the regulation of key amyloidogenic proteins with an emphasis of their aberrant phase separation and aggregation. We also introduce the approaches as potential therapeutic strategies to ameliorate neurodegenerative diseases through intervening protein aggregation. Overall, this review consolidates the research findings of phase separation and aggregation caused by misfolded proteins in a context of neurodegenerative diseases.

## 1. Introduction

A *Lancet Neurology* report published in 2024 indicates that the global burden of neurological diseases is greater than previously predicted based on the systematic analyses the diseases from 1990 to 2021 [1]. According to the available statistical data, the overall burden of neurological disorders has significantly increased from 1990 to 2019, especially for the cases with non-communicable diseases [2]. In 2019 alone, there were globally 805.17 million neurological disorder cases with an age-standardized incidence rate of 10,259.50 per 100,000 population and nearly 10 million deaths [3]. These data illustrate that neurological diseases have become an enormous medical and economic burden across nations and territories.

As progressive neurological disorders, neurodegenerative diseases are characterized by functional defects of neuronal cells and may significantly dampen the life quality of the patients. Many types of neurodegenerative diseases have been reported, and the most common ones include Alzheimer’s disease (AD), Parkinson’s disease (PD), Huntington’s disease (HD), and amyotrophic lateral sclerosis (ALS), among others. Etiologically, most of these diseases are caused by the loss or false function of key regulatory proteins. Despite significant progresses in understanding the pathophysiology and improved treatments of these diseases, many obscurities still need to be further explored and clarified.

Neurodegenerative diseases share many common symptoms, including the impairments of memory, behavior, cognition, sensory and/or motoric functions. Pathologically, most neurodegenerative diseases can be characterized by the aggregation or functional loss of specific proteins [4]. These proteins may share common genetic or cellular mechanisms that contribute to protein aggregation and amyloid plaque formation. Additionally, proteins crucial for different diseases can interact with each other, which may accelerate disease process [5]. Furthermore, significant overlap between the clinicopathological features of many neurodegenerative disorders have been observed, which makes precise diagnosis very difficult [6]. Currently, none of the neurodegenerative diseases has a genuine cure despite significant and innovative research progresses and vast governmental and private investments, and the diseases are eventually fatal.

In a cellular environment, phase separation is a liquid–liquid equilibrium to constrain different biomolecules with relevant activities into membraneless and dynamic niches for specific functional purposes and is a common phenomenon of many proteins to undertake various but specific biological tasks. Phase separation allows the formation of different membraneless compartments or relatively isolated bodies in the cytoplasm and nucleus, such as processing bodies (P bodies), stress granules (SGs), Balbiani bodies, germ granules, promyelocytic leukemia protein (PML) bodies, Cajal bodies, nuclear speckles, and the nucleoli, among others [7]. Typically, biomolecules in normal cellular phase-separated condensates are highly dynamic to efficiently exert their functions, but those in aggregation are immobile and stay in a gel- or solid-like state [8]. Pathologically, protein condensation and aggregation formed by aberrant phase separation contribute to the onset and progression of different diseases [9]. In the past decade, researches from different groups have revealed a causal relationship of protein phase separation and aggregation with the pathogenesis of neurodegenerative diseases, which has extended the mechanistic understanding of these disorders and also provided insights for their therapeutic treatments [10,11]. The exploration of mechanisms, influencing factors, and diagnostic and therapeutic strategies associated with neurodegenerative disorders may contribute to the awareness of the diseases and provide guidance for future exploration and clinical interventions.

In the current review, we aim to summarize the relationship between protein phase separation and aggregation and common neurodegenerative diseases and discuss the latest advances of their treatments.

## 2. Overview of Biomolecular Phase Separation and Aggregation

### 2.1. Definition and Classification of Phase Separation and Aggregation

**Figure 1:**
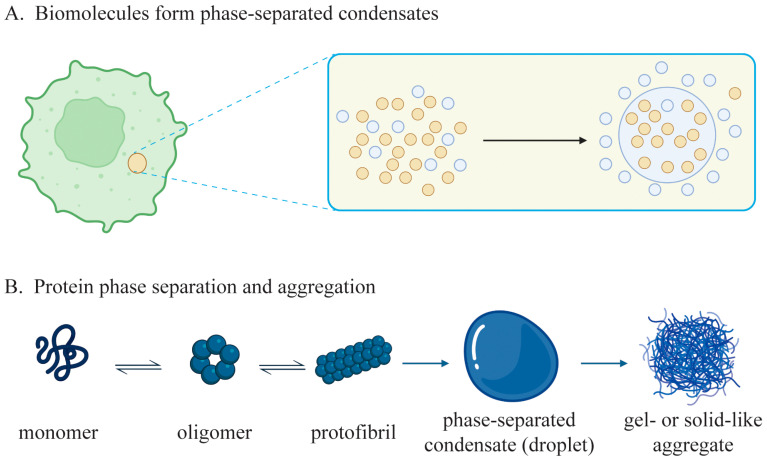
Schematic diagram of biomolecule phase separation and aggregation. (**A**) In a cellular environment, biomolecules regulating a specific process can form phase-separated condensates. (**B**) A protein may form an oligomer, protofibril, and liquid-like droplet. Due to mutations, posttranslational modifications, mislocalization, or other reasons, protein droplets may undergo transition to become gel- or solid-like aggregates.

Phase separation is a type of phase transition in which a system separates into two or more coexisting phases. The formation of separated phases requires a process of phase transition that is a highly coordinated change to break the symmetry in the originally homogenous liquid mixture, leading to the formation of two or more distinct phases [12]. In a binary mixture, the two coexisting phases may include a dense phase and a dilute phase, which is referred as liquid–liquid phase separation in a liquid state. When the concentration of one or more macromolecules exceeds a specific threshold, the solution can separate into two coexisting phases: a dense phase enriched with macromolecules and a dilute phase lacking them [13] (Figure 1A). Protein aggregation is a cellular process to generate relatively large and insoluble aggregates (Figure 1B), which occurs when proteins stay in abnormal structures and/or promiscuously stick together [14].

In neurodegenerative diseases, protein phase separation and aggregation are involved in the formation of aberrant protein inclusions within cells, leading to functional impairment and disrupted regulatory processes, such as cellular metabolism. Aberrant phase separation of proteins can be either reversible or irreversible. For reversible phase separation, condensates can disassociate under specific conditions to restore original protein structures and functions, which requires a liquid status of the condensates and environmental alteration to disrupt the condensation. In contrast, irreversible phase separation forms protein aggregates lacking of the ability to disassociate, resulting in permanent macromolecular inclusions and impaired protein functions [15].

### 2.2. Association between Phase Separation and Neurodegenerative Diseases

Correct folding is essential for cellular proteins to exert their biological functions. Protein folding is regulated by differential intra- and intermolecular interactions, including van der Waals forces, hydrophobic interactions, hydrogen bonds, and electrostatic interactions, among others. Protein misfolding may result from a variety of factors that regulate these interactions, including dominant negative mutations, aberrant posttranslational modifications (PTMs), subcellular mislocalization, and altered environmental factors, such as protein concentration, pH, temperature, and redox condition [16]. Protein aggregation is a common phenomenon in the neuronal cells of neurodegenerative patients, and these aberrant aggregates can be produced either due to misfolding of a mutated protein or an intact endogenous protein that triggers further aggregation. Additionally, divalent cations are considered as molecular glues for proteins [17]. For example, calcium ion (Ca^2+^) can regulate the aggregation of multiple proteins, such as S100 calcium-binding protein A9 (S100A9) and α-syn. S100A9 is an intrinsically amyloidogenic protein and can aggregate together with Aβ and α-syn, which contributes to AD and PD progression, respectively [18,19]. Sanders et al. discovered that the absence of Ca^2+^ allowed S100A9 to exhibit plastic or unstable conformation that favored the onset of fibril formation, while increasing Ca^2+^ concentrations could markedly improve S100A9 stability and consequently reduce its amyloidal aggregation [20]. In contrast, another report indicated that Ca^2+^ binding could induce α-syn structural transition from monomers to extended conformations, leading to its rapid fibrillation and subsequent interfibrillar aggregation [21].

Recent studies have revealed a significant association between phase separation and the pathogenesis of various neurodegenerative diseases. In most cases, functional proteins form fibrous aggregates and lose their activities during neurodegeneration processes. Accumulation of these protein aggregates resulting from aberrant phase separation impairs normal cellular metabolism and functionality, thereby contributing to the onset and progression of these diseases [9].

Neurodegenerative diseases, such as AD and PD, are generally caused by the abnormal phase separation and aggregation of certain proteins, such as tau and α-synuclein (α-syn). These aberrantly phase-separated aggregates exert detrimental effects on neuronal cells and cause cellular damage or even cell death, ultimately resulting in neurodegeneration and cognitive decline. Similarly, in retinal degenerative diseases, such as age-related macular degeneration (AMD), the deposition of phase-separated protein aggregates of lipofuscin and amyloid-β (Aβ) in retinal pigment epithelial cells is closely associated with the disease progression [22]. Furthermore, in muscle degenerative diseases, such as ALS and muscular dystrophy, phase-separated protein aggregation in muscle cells causes muscle damage and functional impairment. Therefore, there is a close association between the aberrant phase separation or aggregation of amyloidogenic proteins and neurodegenerative diseases (Table 1). In-depth mechanistic investigations into the underlying mechanisms linking phase separation and neurodegenerative diseases can shed light on the pathogenesis of these disorders and provide a theoretical foundation for the development of novel therapeutic strategies.

## 3. Types and Characteristics of Common Neurodegenerative Diseases

Neurodegenerative diseases are a class of disorders caused by damage and progressive demise of neuronal cells. Common neurodegenerative diseases include AD, PD, HD, and ALS, among others. These diseases exert different degrees of adverse impacts on the functionality of nervous system and greatly reduce the life quality of patients. In the following sections, we will review the mechanisms underlying protein phase separation and aggregation in these common neurodegenerative diseases and discuss their contributions to the pathogenesis.

### 3.1. Protein Phase Separation and Aggregation in AD

AD is a neurodegenerative disorder characterized by synaptic and neuronal loss in the brains of patients, and subsequently gradual impairment of their cognitive and memory functionality. Common symptoms of the patients include disorientation, declined memory, and language difficulties [40]. In the nerve system of AD patients, Aβ and tau proteins may undergo aberrant aggregation.

**Figure 2:**
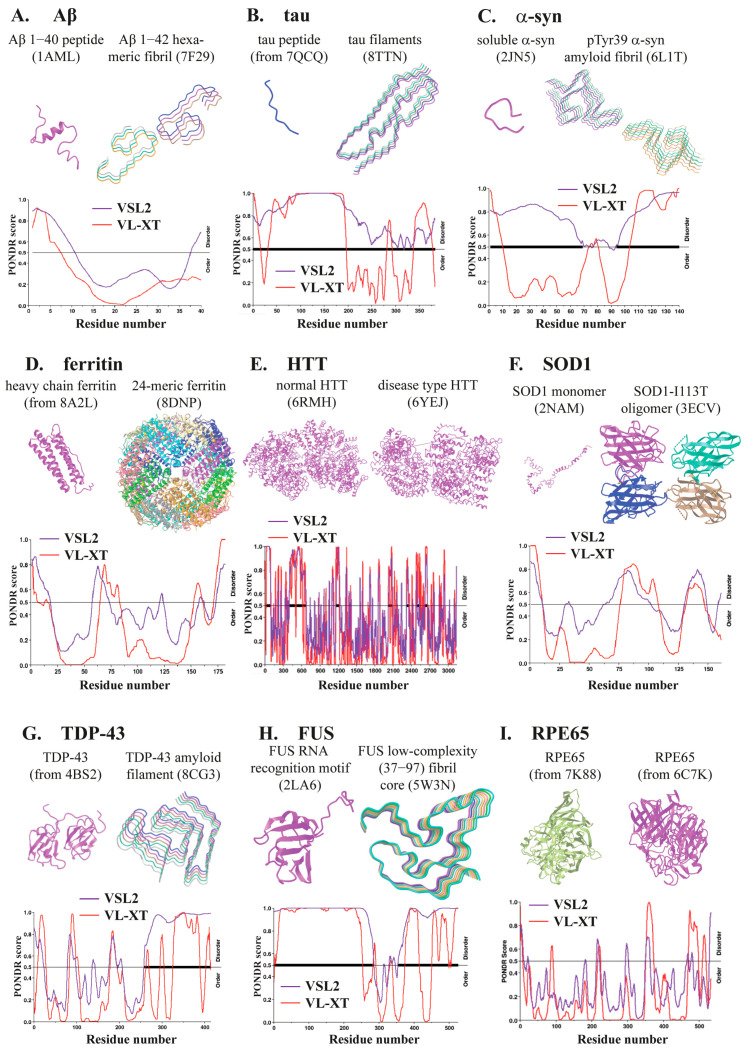
Structures and sequence disorder analyses of key proteins involved in neurodegenerative diseases. From (**A**–**I**), each shows the protein monomer or normal structure (**upper-left** panel), protein oligomer, aggregate, or diseased protein structure (**upper-right** panel), and protein intrinsically disordered region (IDR) analysis using the PONDR algorithm [43] (lower panel). The structure of each protein was obtained from the NCBI Molecular Modeling Database (MMDB), and the PDB ID is provided in parentheses. The word “from” suggests that the image is derived from the PDB ID-corresponded structure by wiping off other interacting molecule(s). For FUS, the structure of whole normal protein is unavailable, likely due to its highly disordered nature, and only the RNA recognition motif is shown. In the IDR analysis, scores higher than 0.5 indicate disorder, and the regions marked by thick lines are recognized as IDRs by the algorithm.

Aβ is produced by sequential proteolytic cleavages of the amyloid precursor protein (APP) mediated by β-secretase (also called β-site APP cleavage enzyme, BACE1) and γ-secretase. Its polymerization into amyloid plaques is one of the characteristic hallmarks of AD and a key pathogenic cause of the disease. Between the two secretases, γ-secretase regulates the final step of cleavage to generate Aβ and is thus considered as a potential target in the treatment of AD [24]. Complex products of Aβ with various lengths from 37 to 43 amino acid residues can be produced by the APP processing, and Aβ42 and Aβ40 are two major peptides generated by γ-secretase. Among these products, Aβ42 is the molecule with the highest liability to form aggregates (Figure 2A) and thus constitutes the major component of amyloid plaques, although it is produced by only 10% of Aβ40 at a molecular ratio.

Phosphorylated tau (Ptau) is another pathological hallmark of AD and can cause neuronal damage and synaptic loss in patients’ brains. As a microtubule (MT)-associated protein, tau protein is predominantly expressed in neurons and possesses multiple functions in healthy brain cells, primarily regulating MT assembly in neuronal cells. However, tau protein may undergo hyperphosphorylation that promotes the formation of tau aggregates (Figure 2B), which are toxic to neurons. Subsequently, the abnormal metabolism of misfolded and aggregated tau proteins in brain regions leads to the formation of neurofibrillary tangles and eventually neurodegenerative disorders, which are generally called tauopathies. Structurally, tau protein has a long intrinsically disordered region (IDR) (Figure 2B), which is a characteristic of many proteins that can undergo phase separation. Importantly, tau protein has over 40 phosphorylation sites. Among these residues, tau phosphorylation at two threonine residues 181 and 217 of tau protein, i.e., Ptau-181 and Ptau-217, is commonly used as pathological markers to assess AD-associated abnormality [25]. Mechanistically, hyperphosphorylation makes tau protein become insoluble and thus detach from MT to form neurofibrillary tangles, which leads to neuronal dysfunction and cell death [41,42].

#### 3.1.1. Effects of Gene Mutations on Protein Phase Separation and Aggregation in AD

Genetic mutations contribute to AD development. *APP* mutations discovered in residues near the cleavage sites of β-secretase and γ-secretase can result in increased levels of Aβ to form aggregates or alter the Aβ42/Aβ40 ratio to produce more toxic Aβ42 peptide. Citron et al. reported that the APP-K670N/M671L mutant could promote the production of total Aβ, leading to increased levels of both Aβ40 and Aβ42 [23]. γ-secretase is a multiunit complex with presenilin-1 (PSEN1) and PSEN2 as components of its catalytic subunit. Mutations of *PSEN1/2* can increase the Aβ42/Aβ40 ratio and consequently cause familial AD with an early-age onset of the disease [44,45,46,47].

The *MAPT* gene encodes the tau protein, and its mRNAs in human have multiple variants due to alternative splicing in its exons 2, 3, and 10, leading to the formation of six protein isoforms with zero, one, or two 2 N-terminal repeats (0N, 1N or 2N) and three or four MT-binding repeats (3R or 4R). Among them, 0N, 1N, and 2N tau isoforms preferentially interacts with other proteins, while 4R isoforms show higher affinity to MT and better ability to promote MT assembly than 3R isoforms [48,49]. Additionally, many mutations in the *MAPT* gene have been reported to exhibit altered activity in tau aggregation, MT assembly, or MT binding. For example, V337M, P301L, and R406W mutants possess significantly increased ability in forming fibrosis aggregation than wild-type (WT) tau. However, V337M mutation can promote the nucleation of filaments, but the R406W mutant shows a reduced rate of this initial polymerization, and the aggregation is quickly accelerated when heparin or arachidonic acid is present. Currently, gaps still exist in understanding how these mutations contribute to tauopathies and what triggers the diseases in the absence of tau mutations [50].

#### 3.1.2. Effects of PTMs on Protein Phase Separation and Aggregation in AD

The major PTMs, their regulators and effects on the aggregation of key amyloidogenic proteins associated with common neurodegenerative diseases are summarized in Table 2. The proteins involved in AD development and progression, such as APP/Aβ, β/γ secretases, and tau, may undergo different PTMs, including phosphorylation, glycosylation, ubiquitination, sumoylation, acetylation, acylation, methylation, and S-nitrosylation [51]. These PTMs impact the aberrant phase-separated or aggregation of these proteins and subsequently alter the disease pathogenesis.

As aforementioned, hyperphosphorylation of the tau protein contributes to neurofibrillary changes of AD. Actually, phosphorylation also regulates the states and activities of other AD-related proteins. Glycogen synthase kinase 3β (GSK3β) and cyclin-dependent kinase 5 (CDK5) have been reportedly to show elevated activity in AD patients’ brains [52,53,54]. GSK3β can primarily phosphorylate T231 of tau, which induces conformational changes to expose its C-terminus for hyperphosphorylation. Multiple phosphatases, such as PP1, PP2A, PP2B, and PP5, can dephosphorylate tau at different phosphorylated serines and threonines.

In addition to tau, BACE1 and APP also undergo differential phosphorylation that regulates BACE1 protease activity, APP-to-Aβ conversion, and Aβ aggregation [55,56]. CDK5 phosphorylates BACE1 at T252 to increase its proteolytic activity, and thus, elevated CDK5 level is associated with AD development [57]. A report by Oliveira et al. demonstrated that Aβ regulates the hyperphosphorylation of APP and tau through promoting the imbalanced kinase and phosphatase activities, which aggravates the symptoms of AD [58].

**Table 2:** Major PTMs, their regulators and effects on protein amyloidal aggregation.

Proteins	PTMs	Modified Sites	Regulators	Effects on Aggregation	References
Aβ (or APP)	Phosphorylation	Thr668, Thr686, Thr654, Ser655, Ser675, Tyr653, Tyr682, Tyr687	GSK-3β, CDK5, ERK1, JNK, ROCK1	↑	[59]
			ROCK2, BACE1, PIN1, heparin	↓	
	Acetylation	Lys132, Lys134	SIRT1	↓	[60,61]
			SIRT2	↑	
	Ubiquitylation	Unidentified	UCHL1	↓	[62]
	Glycosylation	Thr291, Thr292, Ser295, Thr296, Thr576, Thr577, Thr584	GalNAc-T2	↓	[63,64]
			GalNAc-T3	↑	
tau	Phosphorylation	Thr181, Thr212, Ser404, Ser202, Thr231, Thr320	PKA, GSK-3β, Pin1	↑	[65,66]
			PP1	↓	
	Acetylation	Lys280, Lys174	EP300	↑	[67,68]
	Ubiquitination	Lys 48, Lys63, Lys257, Lys259, Lys274, Lys281, Lys321, Lys375, Lys385	CHIP	↑	[69]
			TH006	↓	
α-syn	Phosphorylation	Tyr39, Tyr125, Ser129, Tyr133, Tyr136	CK1, CK2, PLK2	↑	[70,71,72,73]
		Ser87	CK1	↓	[74]
	Ubiquitylation	Met1, Lys6, Lys10, Lys12, Lys21, Lys23, Lys32, Lys34, Lys43, Lys45, Lys96, Lys102	SIAH	↑	[75,76,77]
			CHIP	↓	[78]
	Glycosylation	Thr33, Thr59, Thr64, Thr72, Thr75, Thr81, Ser87	OGT	↓	[79]
	Sumoylation	Lys96, Lys102	HPC2, PIAS2, TRIM28	↑↓	[80,81,82]
HTT	Phosphorylation	Ser13, Ser16	TBK1	↓	[83]
	Methylation	Arg200, Arg205	PRMT4 and PRMT6	↓	[84]
	Ubiquitylation	Unidentified	Ube3a	↓	[85]
SOD1	Phosphorylation	Thr2	ATM/CHK2, ATR/CHK1	↓	[86,87]
	Acetylation	Unidentified	HDAC6	↓	
	Sumoylation	Lys75	SUMO1, SUMO3	↑	
TDP-43	Phosphorylation	Ser48, Ser379. Ser403, Ser404, Ser409, Ser410	CK1, CK2, CDC7, TTBK1, TTBK2	↑	[88]
	Ubiquitination	Unidentified	Parkin	↑	[89]
FUS	Phosphorylation	Tyr526	Abl	↑	[90]
	Acetylation	Lys510	HDACs	↓	[91]

Note: ↑ and ↓ depict promoting and inhibiting aggregation, respectively.

Based on the functional impacts of phosphorylation on the aggregation of AD-associated proteins, the intervention of specific kinase and phosphatase activities is theoretically a practical and effective strategy to ameliorate AD symptoms.

### 3.2. Protein Phase Separation and Aggregation in PD

PD is a chronic and progressive neurodegenerative disorder characterized by a deficiency of dopamine neurons, which leads to various symptoms including muscle stiffness; tremors; impaired movement; and difficulty in speaking, swallowing or chewing; among others. In the pathogenesis of PD, the disorders of two key proteins, α-syn and ferritin, play significant roles.

The α-syn protein is encoded by the *SNCA* gene and regulates neuronal transmission through synaptic vesicle trafficking. In PD patients, α-syn forms insoluble amyloid fibrils enriched with β-sheets (Figure 2C) that are major components of Lewy bodies and interfere with normal cellular functions by causing neuronal damage or even cell death [26]. Based on the primary protein sequence, the α-syn protein has an IDR at the C-terminus (Figure 2C). Consistently, α-syn can form phase-separated droplets, and the hardening process of these droplets has been associated with the transition to PD pathogenesis [92]. A recent study by Agarwal et al. revealed that the negatively charged C-terminus of α-syn regulates its phase separation through interacting with its binding partner VAMP2, Ca^2+^, or spermine, and this process depends on α-syn association with lipid membrane [93]. Therefore, the α-syn protein has a highly foldable structures and may undergo conformational changes under specific conditions, making it prone to aggregation. Thus, environmental factors can also contribute to α-syn aggregation and PD development. As a chemical inducer of PD-like symptoms, rotenone can promote the formation of structurally α-syn oligomers and fibrils that potentiate the oligomer formation in adjacent neuronal cells [94].

PD development is associated with an abnormal aggregation of brain iron protein. Excessive iron has been causally linked to the generation of oxidative stress, which causes degeneration in neuronal cells. Deposition of iron ions in PD patients, particularly in regions with high dopamine neurons like substantia nigra, promotes the progression of the disease. In a study by Shi et al., iron accumulation was observed to correlate with degeneration of dopamine neurons and motor deficits in monkeys with drug-induced PD [95].

Interestingly, α-syn can act as a ferrireductase to reduce iron from Fe^3+^ to Fe^2+^ with copper as a cofactor [96]. However, it is still not completely clear how the reductase activity of α-syn is associated with PD pathogenesis. One possibility is that increased α-syn expression results in its protein aggregation and subsequent loss of ferrireductase activity, leading to accumulated Fe^3+^ and decreased Fe^2+^. Since Fe^2+^ is generally needed by various cellular reactions, its insufficiency may adversely impact the survival and functionality of dopaminergic neurons. Alternatively, increased α-syn expression could lead to the excessive production of Fe^2+^, which may interact with other cellular proteins or lipids and produce radicals through the Fenton reaction to generate toxic products in neurons [96]. McDowall et al. detected a significantly reduced ferrireductase activity in PD patient brains, supporting the hypothesis that the deposition of inactive α-syn protein is a major etiologic cause for the neuron loss of the disease [97].

As an essential iron storage protein, ferritin is a large multiunit spherical molecule containing 24 subunits (Figure 2D) that are consisting of two types of peptides, H-ferritin (FTH), and L-ferritin (FTL). With tissue specificity, FTH and FTL subunits may have different ratios to build the ferritin shell that can store over 4000 Fe^3+^ ions [28]. FTH, but not FTL, possesses ferroxidase activity to convert Fe^2+^ to Fe^3+^ iron, prior to iron storage in the ferritin core [98]. Aberrant iron deposition in the brain is associated with various neurodegenerative diseases, including PD, AD, HD, and ALS [99,100]. The oxidative stress caused by accumulated iron can generate hydroxyl radicals through the Fenton reaction and subsequently contribute to the pathogenesis of PD. Iron accumulation in the brain is also the phenomenon of another neurodegenerative disease, neuroferritinopathy, which is inheritable and often diagnosed with PD and AD in older patients [29]. Ferritin can both undergo phase separation and form aggregates in brain [29,101], but its pathological relevance to PD development has not been elucidated.

#### 3.2.1. Effects of Gene Mutations on Protein Phase Separation and Aggregation in PD

The α-syn protein may form fibrils due to mutations of the *SNCA* gene, altered protein conformation, or increased expression. Many familial mutations, such as point mutations at the N-terminus and truncation of C-terminal region, are associated with an increased risk of PD [102,103]. In a patient with a familial history of PD, an H50Q hereditary mutation of the *SNCA* gene was discovered, and the mutated protein α-syn-H50Q showed two special polymorphs with narrow and wide fibrils formed from one or two protofilaments, respectively. The H50Q protein in both polymorphs exhibited accelerated aggregation kinetics, high seeding capacity, and increased cytotoxicity compared to WT α-syn [104,105]. Clinically, the *SNCA* gene locus duplication and triplication have been determined as the cause of PD development and linked to the disease [106,107]. Recently, Yang et al. reported a 21-nucleotide duplication in the exon 2 of one *SNCA* gene allele. This genetic alteration could produce an MAAAEKT peptide insertion at the N-terminal region and cause rapid PD development, with abundant α-syn positive pathology observed throughout the cerebral cortex of the patients [103].

The accumulation of oxidative substances and disruption of protein degradation pathways in PD patients contribute to α-syn aggregation [108]. The *GBA1* gene encodes the lysosomal enzyme glucocerebrosidase, and its deficiency, caused by mutations, leads to accumulation of glycolipid substrates, which trigger neuronal inflammation and α-syn aggregation to promote PD progression. Individuals with *GBA1* mutations exhibited up to 30-fold increased risk of PD development and early-age onset of the disease [27]. When these degradation pathways are impaired, α-syn may form aggregates in neurons [109]. Meanwhile, α-syn also induces Aβ secretion and amyloid production through promoting β-/γ-secretase-mediated processing of APP [94,110].

#### 3.2.2. Effects of PTMs on Protein Phase Separation and Aggregation in PD

For PD-related PTM studies, most research efforts have been devoted to α-syn, which may undergo various modifications, such as phosphorylation, ubiquitination, glycosylation, and sumoylation [111]. The PTMs of other PD-related proteins, such as FBXO7, HTRA2, VPS35, and DJ-1, have also been reported [112].

In 2000, Okochi et al. reported that α-syn in rat pheochromocytoma cells was constitutively phosphorylated with a primary target site of Ser129, mediated by CK1 and CK2 [72]. Another study indicated that Polo-like kinase 2 (PLK2) could also phosphorylate α-syn at Ser129 in the central nervous system [73]. Lu et al. identified multiple phosphorylated tyrosines in α-syn and revealed that phosphorylation at Tyr125 and Ser129 could alter its binding affinity to metal ions [113]. When synthetic α-syn fibrils were introduced into primary-cultured neurons, they could act as seeds to induce α-syn aggregation by converting normal α-syn monomers into phosphorylated aggregates, which could be recapitulated in the brains of a mouse model. When the α-syn aggregates were injected into one side of the brain, phosphorylated α-syn pathology could also be detected on the contralateral side [114]. Consistently, Awa et al. revealed that these extracellular seed-induced phosphorylation of endogenous α-syn could appear in a few days post injection, locate at the pre-synaptic region initially, and then spread to the cell body [115]. Interestingly, phosphorylation at different sites of α-syn may have differential effects on α-syn aggregation. Ser87 phosphorylation of α-syn, which could also be promoted by CK1, reportedly increased its conformational flexibility, inhibited oligomerization, and blocked fibrillization [74].

Other PTMs of α-syn also impact its aggregation. For example, accumulation of ubiquitinated α-syn has been recognized as a pathologically toxic factor in the brains of PD patients [112]. The injection of synthetic α-syn fibrils into mouse brains could also induce the accumulation of ubiquitinated α-syn [114]. Consistently, α-syn ubiquitination mediated by SIAH1 could promote its aggregation and cell apoptosis [76]. Another E3 ligase, Parkin, could also promote the ubiquitination of α-syn in human brain, but its pathological role in PD development was undetermined [116]. As a type of glycosylation, O-GlcNAcylation of α-syn generally inhibited α-syn aggregation without altering its membrane-binding affinity [79]. Furthermore, sumoylation exhibited differential effects on α-syn aggregation. In α-syn aggregates of PD patients’ brains, increased sumoylation could be detected, and several studies have indicated that sumoylation could promote α-synuclein pathology [80]; however, other studies have still suggested that sumoylation could relieve α-syn aggregation induced by methamphetamine treatment [81], and non-sumoylated isoforms of α-syn were more aggregation-prone and toxic than other α-syn variants [82].

### 3.3. Protein Phase Separation and Aggregation in HD

HD is an inherited disorder characterized by motor and cognitive impairments that typically arise in adulthood [117]. In this disease, dominantly inherited mutations in exon 1 of the *HTT* gene generate mutant huntingtin (*mHTT*) with abnormal expansion of a CAG-repeated stretch, which leads to an increased number of glutamine residues to form polyQ-expanded HTT proteins. The CAG expansion causes abnormal HTT protein aggregation (Figure 2E), leading to incurable neurodegenerative disorders [118].

#### 3.3.1. Effects of Gene Mutations on Protein Phase Separation and Aggregation in HD

HTT is a large protein with 3,144 amino acids encoded by the *HTT* gene and is normally involved in neuronal functions, such as cellular signaling and axonal transport [30]. Multiple studies have indicated the involvement of phase separation and phase transition in the formation of mHTT inclusion [119,120]. The C-terminal region contains IDRs (Figure 2E). Consistently, HTT may undergo liquid–liquid phase separation promoted by the weak hydrophobic interaction of its N-terminal PolyQ and proline region, which can be converted into solid-like assemblies that initiate irreversible pathological aggregation [119].

Excessive PolyQ regions alter the structure of the HTT protein, increasing the liability to form small aggregates (Figure 2E) that further generate Huntington’s bodies, a major pathological feature of HD [31]. Based on the report of Peskett et al., the cleavage of mHTT released the PolyQ-containing N-terminal amphipathic fragments that can form reversible liquid-like condensates and then transform into solid-like fibrillar assemblies both in vitro and in cells [119]. HTT can separate into different phases depending on their concentrations. Posey et al. proposed three different phases formed by N-terminal fragment of HTT, including M, S, and F phases, which are composed of soluble monomers and oligomers, bigger soluble aggregates, and insoluble fibrillar aggregates, respectively [121].

Caron et al. observed that mHTT formed loosely packed globular inclusions and tightly packed fibrillar aggregates with or without protein exchange with the soluble phase, respectively [122]. Ramdzan et al. proposed a model of HD progression: nascent aggregates of disordered HTT proteins undergo maturation to form amyloid inclusions that sequestrate soluble HTT, leading to reduced apoptosis, decreased cell metabolism, and the slow death of neuron cells [123]. Additionally, mHTT aggregates can be formed in both the nucleus and cytoplasm, which may cause neuronal cell toxicity through different mechanisms [124]. The accumulation of these aggregates exerts toxic effects on neuronal cells. They can trigger oxidative stress and impair mitochondrial functions, gradually causing neuronal damage and cell death, with more pronounced effects in voluminous regions of brains, such as the striatum. The synaptic function of HD patients can also be affected, with attenuated neurotransmitter release and synaptic plasticity that further impair neuronal communication and network integrity.

Some studies have suggested that the HTT protein may influence the formation and function of nucleoli, and its abnormal conformation may alter nuclear localization. Mutated HTT proteins may interfere with ribosomal RNA (rRNA) synthesis in nucleoli and affect the assembly of nucleolar proteins and nuclear pore complexes within the nucleoli, thereby adversely impacting nucleolar formation and function [118,125]. All these can meddle the regulatory network of gene expression, leading to accelerated pathological process of HD.

#### 3.3.2. Effects of PTMs on Protein Phase Separation and Aggregation in HD

Different PTMs exert differential effects on HTT protein solubility and its aggregation. The N-terminal region containing the polyQ can form intracellular inclusions with higher toxicity than the full-length HTT mutant [126]. DeGuire et al. reported that phosphorylation at Ser13 and Ser16 inhibited protein aggregation caused by the expression of the HTT N-terminal region encoded by its exon 1 (*HTT*-exon1). Their further investigation revealed that phosphorylation acts as a molecular switch to regulate HTT aggregation, helical conformation, and internalization, suggesting that manipulating HTT phosphorylation is a potential strategy to intervene HD progression [127]. On the contrary, acetylation of HTT at the lysines within the first 17 residues promotes the aggregations of HTT and HTT-exon1 proteins [128,129]. Additionally, arginine methylation, especially at R200 and R205 mediated by PRMT4 and PRMT6, promotes HTT phase separation and increases its solubility, showing beneficial effects on neuronal survival [84].

A study by Bhat et al. reported that HTT may undergo differential polyubiquitination through K48 and K63 in ubiquitin that can either promote HTT proteasomal degradation or accelerate its aggregation, respectively [85]. Similar to the latter scenario of undegradable ubiquitination, sumoylation has also been reported to promote *HTT*-exon1-encoded protein forming relatively large and amorphous aggregates [130].

### 3.4. Protein Phase Separation and Aggregation in ALS

Degenerative muscle diseases are acquired or inherited neuromuscular disorders of the anterior horn cells, peripheral nerves, neuromuscular junctions, and muscle that cause muscle degeneration and weakness [131]. The most common muscular degenerative disease is ALS, characterized by muscle weakness, atrophy, and progressive limb motor impairment [132]. Multiple mechanisms may lead to ALS pathogenesis, including oxidative stress, mitochondrial dysfunction, endoplasmic reticulum stress, aberrant RNA metabolism, and neuroinflammation, among others [133].

In ALS, the major proteins involved in the disease development are superoxide dismutase 1 (SOD1), TAR DNA-binding protein 43 (TDP-43), and Fused in Sarcoma (FUS) [35,134,135].

SOD1 protein generally forms homodimer that can break toxic superoxide radicals into hydrogen peroxide and oxygen. Each SOD1 monomer has eight anti-parallel β-strands that form a β-barrel structure with a hydrophobic core [136]. SOD1 is an antioxidant protein associated with Cu and Zn ions essential for its catalytic activity and structural stability, respectively. Two IDRs in monomeric SOD1 form the electrostatic loop and Zn loop that determine its biological function and structural stability [137] (Figure 2F). SOD1 possesses high propensity to form aberrant aggregates in astrocytes and microglia under conditions of oxidative stress or mutations, leading to the development of ALS [32].

SOD1 aggregation is generally detected in stress granules that are membraneless organelle formed by proteins and nucleic acids through the phase separation mechanism. Two recent studies have described phase separation as a prelude of SOD1 aberrant aggregation [138,139]. A recent study by Das et al. revealed that Zn depletion can trigger structural distortion of the two loop regions and subsequently promote SOD1 phase separation and amyloid formation. Consistently, Zn addition can stabilize the loops and reduce SOD1 phase separation and aggregation. However, the presence of Zn may only prevent the formation of SOD1 accumulation or ALS onset, but cannot resolve SOD1 inclusion or mitigate the disease, because its addition does not reverse the aggregated state of SOD1 [138].

TDP-43, also called TARDBP, is a transcriptional factor and also an RNA-binding protein with a total of 414 amino acids that contains two highly conserved RNA recognition motifs. The C-terminal region of TDP-43 is a long IDR with 141 amino acids (Figure 2G), which is also called the prion-like domain (PrLD) and determines the phase separation ability of TDP-43 [140]. In healthy cells, TDP-43 stays in the nucleus regulating RNA metabolism [141]. In glial cells of the gray and white matter spinal cords of ALS patients, TDP-43 can translocate from the nucleus to the cytoplasm where it forms cytoplasmic aggregates and inclusions [33,34]. Stimulated by different mechanisms, TDP-43 exhibits aberrant phase separation and accumulates as non-dynamic stress granules, leading to pathological development of ALS.

FUS is an RNA binding protein consisting of 526 residues and contains multiple functional domains, including a PrLD, an RNA recognition motif, a zinc finger domain, and a Gly-rich domain, among others. The FUS protein is highly disordered, especially at its N-terminal region containing the PrLD (Figure 2H). FUS is mostly a nuclear protein in neuronal cells but can shuttle between the nucleus and cytoplasm [142]. As a pleiotropic protein, FUS has been reported to regulate transcription by its N-terminus, RNA processing by its C-terminus, and DNA repair [35].

FUS mislocalization from the nucleus to the cytoplasm and the formation of its cytoplasmic inclusions are the characteristic phenomena of ALS pathology. Yang et al. demonstrated that subcellular localization and RNA binding regulated FUS architecture, independent of its WT or mutant status. The nuclear FUS formed oligomers in a granular shape, but the cytoplasmic FUS stayed in inclusions without detectable oligomerization. The formation of both the nuclear oligomers and cytoplasmic inclusions of FUS depended on its RNA binding [143].

#### 3.4.1. Effects of Gene Mutations on Protein Phase Separation and Aggregation in ALS

In 1993, Rosen et al. first reported 11 different SOD1 missense mutations in 13 families with familial ALS [135]. Currently, over 180 natural mutations of SOD1 associated with familial ALS have been reported [144].

Berdynski et al. analyzed 915 Polish ALS patients and identified 15 SOD1 mutations in 21.1% familial and 2.3% sporadic ALS cases. Their data supported the notion that accumulation of misfolded aggregates but not the loss of functional protein contributes to the pathogenicity of SOD1 mutations [145,146]. Among different SOD1 mutations, L144S was significantly associated with the longest overall survival and the least severity, while G41S correlated with the shortest survival and the most aggressive ALS progression [145].

As mentioned above, Zn ion stabilizes SOD1 to prevent its phase separation and amyloid formation. Many of reported SOD1 mutations disrupt its disordered electrostatic loop, while others may attenuate its Zn binding ability [138]. SOD1-G85R loses most Zn affinity, likely due to the proximity of mutation to Zn-binding domain, and only binds about 5% Zn compared to WT SOD1 [147]. Thus, exogenous Zn only partially reduces SOD1-G85R aggregation [138]. However, for another SOD1 mutant I113T with attenuated Zn binding caused by mutational stress of protein dimeric surface, its phase separation could be attenuated by Zn in a dose-dependent manner. SOD1-G37R is a mutant with Cu-binding deficiency and does not undergo phase separation in the presence of metal ions. However, under metal-free conditions, SOD1-G37R also formed phase-separated condensates, which could be disrupted by the addition of Zn but not Cu, suggesting an essential role of Zn in modulating SOD1 phase separation and amyloid formation in ALS patients [138]. Clinically, ALS patients with SOD1-G37R mutations exhibit a less severe phenotype than SOD1-G85R and -I113T mutations [138].

Most TDP-43 mutants exhibit deficiency in proper phase separation, leading to structural instability and aberrant protein aggregation. Consistently, the majority of ALS-associated TDP-43 mutations are located in the C-terminal PrLD [148]. TDP-43-A315E and -A315T mutations have been associated with the onset of familial ALS [149,150]. Liu et al. employed microsecond all-atom molecular dynamics simulations to investigate the early aggregation process of TDP-43 and revealed distinct mechanisms of the two mutants in inducing the irreversible aggregation of the TDP-43 (312-317) peptide [151]. A more recent study by the authors indicated that A315T and A315E mutations increased inter-molecular interactions and promoted β-barrel formation of TDP-43 (307-319) hexamer, which provided mechanistic insights to the understanding of enhanced neurotoxicity by A315 mutations [152]. Sreedharan et al. used chick embryos to express TDP-43-Q331K and -M337V mutants and observed that both mutants exhibited dramatic retardation of developmental maturation with defects to develop normal limbs and tails, and a significant increase of apoptotic cells compared to embryos bearing WT TDP-43 [153]. Johnson et al. also demonstrated that six different mutations in the C-terminal disordered region accelerated the aggregation propensity and exhibited increased toxicity to neuronal cells, linked to the degenerative neurons of ALS patients [154].

*FUS* gene mutations are associated with the development of type 6 ALS. Most *FUS* mutations could lead to its mislocalization, which promotes the formation of cytoplasmic FUS inclusions [155]. Waibel et al. reported that the R495X mutation of *FUS* created a premature stop codon and subsequently deleted its nuclear localization signal at the C-terminus. The truncated FUS protein stayed in the cytoplasm to form aggregated inclusions and caused a severe disease phenotype [156]. Mechanistically, FUS-R495X could induce neurotoxicity by reducing mitochondrial sizes, changing the morphology and attenuating their function [157]. Strikingly, ALS patients with *FUS* mutations typically manifest the first symptoms before 45 years of age, which is significantly earlier than the individuals bearing other types of ALS [158].

#### 3.4.2. Effects of PTMs on Protein Phase Separation and Aggregation in ALS

The SOD1 protein stays as a homodimer, with each subunit forming a β-barrel structure that is stabilized by the C57-C146 disulfide bond with a zinc ion in its active site [145]. The initial step of SOD1 misfolding and aggregation is its homodimer dissociation, and the SOD1 monomers are highly sensitive to the loss of zinc ion that stabilizes the structure. Thus, dissociation of the SOD1 homodimer is a critical step for its aggregation [159,160]. Cys111 is a residue close to the tripeptide moiety of SOD1 homodimer interface, and its glutathionylation is abundant in human tissues [161]. Redler et al. reported that Cys111 glutathionylation destabilized SOD1 homodimerization and subsequently promoted its aggregation, leading to ALS development [162].

Multiple studies have suggested that peripheral blood mononuclear cells (PBMCs) are involved in triggering degenerative process of motor neurons [163,164,165]. In PBMCs of sporadic ALS patients, SOD1 stays as aggregates in the cytoplasm, which contributes to increased DNA damage. In PBMCs of healthy individuals but not ALS patients, the ATM/CHK2 and ATR/CHK1 pathways can be activated, leading to SOD1 phosphorylation that allows its translocation from the cytoplasm to the nucleus to protect DNA from oxidative damage [166]. Consistently, Fay et al. reported that an SOD1-T2D mutant, which mimics Thr2 phosphorylation, could stabilize SOD1 homodimer without affecting its native structure and subsequently prevent the formation of toxic aggregates. The mitigative effects of phosphorylation on SOD1 aggregation could be extended to A4V, a prevalent SOD1 mutant causing aggressive ALS phenotype [167].

TDP-43 may also undergo differential PTMs. Neumann et al. discovered hyperphosphorylation and ubiquitination of cytosolic TDP-43 aggregates in post-mortem samples of ALS patients [134]. Hasegawa et al. generated 39 antibodies to analyze potential phosphorylation sites in TDP-43 and identified multiple phosphorylated residues, including serines 379, 403, 404, 409, and 410, in the C-terminal regions of aggregated TDP-43. Phosphorylation of TDP-43 increased its propensity of oligomerization and fibrillization, and it contributed to pathogenesis of ALS [168]. Among these phosphorylation sites, immune detection using antibodies against Ser409 and Ser410 phosphorylation is a golden standard to evaluate aggregated TDP-43 inclusions in sporadic and familial ALS [169].

Increased TDP-43 ubiquitination can deplete free ubiquitin pool, while aberrant TDP-43 accumulation may sequester the components of ubiquitin-proteasome system and attenuate proteasome activity. The overall consequence is the perturbation of ubiquitin homeostasis and accumulation of polyubiquitinated proteins [170,171]. Rayner et al. reported that the E3 ligase SCFcyclin F complex could promote the ubiquitination of TDP-43 [172].

The most studied PTMs of FUS are Ser/Thr phosphorylation and Arg methylation. At least 32 Ser or Thr residues have been reported as putative phosphorylation sites, but most of them were determined based on mass spectrometry studies [173]. Deng et al. reported that DNA-PK-mediated phosphorylation in the PrLD of FUS was a response to DNA damage and could promote its cytoplasmic translocation [174]. Other researchers have also reported that FUS accumulated at the DNA damage sites and FUS depletion could impair the double-strand break repair process [175,176]. Consistently, Monahan et al. observed that phosphorylation in the PrLD could decrease aggregative propensity of FUS, disrupt its aggregation, and reduce its cytotoxicity [177,178]. Another report also demonstrated that Abl kinase could mediate FUS phosphorylation at Tyr526, which promoted its cytoplasmic translocation and toxic aggregate formation [90].

FUS has been shown to be mono- and/or dimethylated by protein arginine methyltransferase 1 (PRMT1) and PRMT8 [179]. Asymmetric dimethylation of Arg sites could reportedly promote FUS-mediated transcriptional activation [180]. The nuclear localization signal (NLS) of FUS is located within the third Arg-Gly-Gly region at its C-terminus, in which several Arg residues decide its nuclear localization. Dimethylation of these Arg residues was associated with FUS translocation to cytoplasm, while Arg demethylation restored its nuclear localization [181,182]. Additionally, mono- or unmethylated arginines could enhance FUS nuclear retention [183]. Thus, differential Arg methylation modulates distributions of FUS between the nucleus and cytoplasm.

Overall, PTMs, especially phosphorylation, may exert differential effects on SOD1 and TDP-43. Phosphorylation favors SOD1 homodimerization that prevents its aggregation and ALS progression but facilitates the formation of TDP-43 inclusions that promote the pathological process. Noticeably, initial filaments of a TDP-43 mutant possess seeding activity to promote the secondary nucleation and aggregation of non-pathological TDP-43 protein, which impairs neuronal cells and aggravates ALS development [184].

### 3.5. Protein Phase Separation and Aggregation in AMD

Retinal degenerative diseases are characterized by the progressive degeneration and demise of retinal cells. Their most prevalent types include AMD and diabetic retinopathy (DR), which may result in declined vision and even blindness [185].

AMD is a common age-related eye disease that typically affects individuals over the age of 50. It primarily impacts the macular region of retina, leading to central vision loss. The development of AMD is influenced by factors including inflammation, gene mutations, abnormal lipid metabolism, and alterations in the extracellular matrix of Bruch’s membrane and retinal pigment epithelium (RPE) layer [186]. In early stages of AMD, thickening of Bruch’s membrane in the macular region may attenuate nutrient supply and accelerate disease progression. In AMD, Aβ and retinoid-associated proteins may undergo abnormal aggregation [187,188].

As discussed above, Aβ aggregation is primarily associated with AD, but its deposition has also been observed in the retinas of AMD patients, suggesting its involvement in retinal pathogenesis. Additionally, the aggregation of retinoid-associated proteins, especially retinal pigment epithelium 65 kDa (RPE65), plays a role in macular impairment in AMD patients [187]. RPE65 is a retinoid isomerohydrolase in RPE cells, converting all-trans-retinyl esters into 11-cis-retinol for visual pigment regeneration in photoreceptor cells [38,189]. Its aggregation and deposition may impair normal activity, trigger macular degeneration, and promote the development of AMD. Mechanistically, RPE65 has over 100 mutations with pathogenic consequences [190]. Aggregation of retinoid-associated proteins may also be linked to dysregulation in retinoid metabolism pathways [191]. Abnormal aggregation and deposition of Aβ and RPE65 proteins result in macular damage and degeneration, leading to central vision loss, distortion, and difficulties in dark adaptation of AMD patients [187,188]. Exploration of molecular mechanisms underlying AMD is an active area of research and may improve understanding and develop new treatment strategies.

#### 3.5.1. Effects of Genetic Variations on Protein Phase Separation and Aggregation in AMD

RPE65 protein is an enzyme to produce 11-cis-retinol essential for normal vision. The D477G mutation of RPE65 was first discovered in 2011 in two Irish families and caused autosomal dominant retinitis pigmentosa with the involvement of choroid and macula [192]. This mutation increases RPE65 aggregation propensity by generating an aggregation-prone surface (Figure 2I), and its dominant-negative effect is likely due to the dimerization between D477G and WT RPE65, leading to mislocalization of the WT protein [39].

Apolipoprotein E (ApoE) belongs to the lipoprotein family of cholesterol transporters and is involved in cholesterol metabolism and immune modulation. In humans, it exists in three polymorphism isoforms, ApoE2, ApoE3, and ApoE4, that show differences at the positions 112 and 158 (Cys112-Cys158 for ApoE2, Cys112-Arg158 for ApoE3, and Arg112-Arg158 for ApoE4) [193]. Among them, the APOE2 allele is a risk factor for AMD development but protective against AD, while the APOE4 allele protects its carriers against AMD but increases the risk of AD [36,37]. A study by Cunza et al. demonstrated that ApoE2 allele could cause inefficient cholesterol transport and subsequently increase RPE ceramide. This could lead to mitochondrial injury that promotes ApoE2 phase separation mediated by the thiol oxidation of cysteines at the 112 and 158 positions. These condensates are the potential precursors of drusen that are yellow lipid and protein deposits under retina and causally associated with AMD. However, ApoE4 lacking these two cysteines is resistant to this phase-separated condensation. Thus, ApoE2 protein phase separation caused by mitochondrial stress is an important pathogenic mechanism and may serve as a therapeutic target in AMD [194].

#### 3.5.2. Effects of Dysregulated Metabolism on Protein Phase Separation and Aggregation in AMD

Aggregation of retinoid-associated proteins may also be linked to dysregulation in retinoid metabolism pathways [191,195]. In retina, retinol, a type of retinoid, is transported into RPE cells through retinoid-binding proteins and then metabolized into retinoid products.

The abnormal aggregation and deposition of Aβ and RPE65 proteins result in damage and degeneration in the macular region, ultimately leading to symptoms including central vision loss, distortion, and difficulties in the dark adaptation of AMD patients [187,188]. Currently, exploration of molecular mechanisms underlying AMD is still an active and fertile research area to improve our understanding and create new treatment strategies of this disease.

### 3.6. Protein Phase Separation and Aggregation in DR

DR is a retinal degenerative disease and commonly occurs in patients with a long term of diabetes [196]. The disease can result in retinal vessel damage, bleeding, and ultimately blindness. The complex mechanisms of DR involve various signaling pathways and biological processes, including high glucose-induced oxidative stress, inflammation, advanced glycation end products (AGEs), angiogenesis, vascular permeability, increased cell apoptosis, and the activation of various signaling pathways [197,198,199]. In DR patients, AGEs can undergo abnormal accumulation in photoreceptor cells.

AGEs that are commonly generated during hyperglycemia intricately bind to proteins within photoreceptor cells and form complexes that accumulate in the cells. Interacting predominantly with receptors for advanced glycation end products (RAGE) on the cell surface, AGEs initiate cascades of signaling pathways that can promote inflammation, oxidation, and other reactions adverse to cellular activities. AGEs can accumulate in the extracellular matrix, causing the crosslink of matrix proteins, structural distortion, and functional impairment of retina [200]. Current research of aggregated AGEs prominently focuses on DR, but investigation into whether classical phase separation occurs in AGEs remains relatively limited. AGEs may clearly engage with regulatory proteins or other molecules, forming intricate structures, yet the precise mechanism of phase separation awaits future exploration.

### 3.7. Contributions of RNA Binding Proteins to Neurodegenerative Diseases

RNAs are highly versatile molecules undertaking many regulatory functions in cells. Eukaryotic cells contain numerous RNA-binding proteins (RBPs) that bind or cover various RNAs to regulate the processes of their biogenesis, splicing, transportation, translation, and stability, among others. RBPs associate with RNA partners to form ribonucleoproteins (RNPs) that possess diverse activities [201]. Among over 1500 RBPs with about 600 structurally distinct RNA binding domains (RBDs), each protein may contain one or more RBDs [201]. Based on binding targets and structures, RBDs can be classified as an RNA recognition motif (RRM), Gly/Arg-rich (RGG) motif, zinc finger (ZnF) domain, S1 domain, K-homology (KH) domain, and double-stranded RNA-binding domain (dsRBD) [202]. Mechanistically, an RNA molecule may generate specific sequence motifs to guide RBPs for their functional execution or bear specific recognition elements to serve as a scaffold or assembly platform for RBP recruitment and their orchestrated actions [201,203].

A large portion of RBPs contains extended IDRs that confer these proteins with the ability to form phase-separated condensates knitted by protein–protein and RNA–protein interactions [204,205]. Some RNP granules contain amyloid-like structures that can form either biomolecular condensates or amyloid-like fibrils [13,206]. Many RBPs play a crucial role for neuronal functions through delivering specific mRNAs to designated subcellular compartments of neuronal cells in axons and dendrites for their localized translation and protein synthesis. Thus, gene mutations or deletions that functionally disrupt RBPs may cause various neurological disorders, such as ALS and spinal muscular atrophy (SMA) [207].

The *SMN1* gene encodes the full-length SMN protein, which is homozygously deleted in SMA, characterized by the selective destruction of α-motor neurons in spinal cord [208]. The *SMN2* gene is a duplicate of *SMN1* but has a C6U point mutation in its exon 7. This mutation causes poor recognition of the exon during splicing and subsequent predominant exon 7 skipping, which produces an unstable SMN protein. As a ubiquitously expressed RBP, SMN is essential for many RNA-related processes, including structure formation, biogenesis of small nuclear RNPs (snRNPs), RNA splicing, transport, and assembly [209,210]. Thus, *SMN* deficiency increases the propensity of neurodegenerative disorders. In a mouse model with human SMN2 expression at a *Smn*-null background, the mice manifested about 35% loss of spinal cord and up to 40% loss of the facial nucleus [211].

The Chromosome 9 Open Reading Frame 72 (*C9ORF72*) gene is the most commonly mutated gene in ALS and frontotemporal dementia (FTD). Previous studies have indicated that C9ORF72 regulates actin dynamics and endosomal recycling of glutamate ionotropic receptor AMPA type subunit 1 (GRIA1) in neuronal cells at the synapse. Additionally, C9ORF72 also regulates autophagy initiation, substrate recruitment for autophagy degradation, and autophagosome maturation and closure [212].

During the pathogenesis of ALS and FTD, the 5′-UTR of the *C9ORF72* mRNA contains markedly expanded hexanucleotide (GGGGCC or G4C2) repeats (typically over 30 versus less than 25 in normal individuals). The presence of G4C2 repeats or DNA G-quadruplexes formed by these G-rich sequences can attenuate *C9ORF72* transcription and subsequently reduce its expression. Multiple G4C2 repeats in the genome may also initiate bidirectional transcription of this region to produce G4C2 sense- and G2C4 antisense-expanded transcripts, which form G-quadruplexes (a noncanonical secondary nucleic acid structure) or hairpins. RNA molecules enriched with these structures may generate RNA foci that sequester different RBPs. The RNAs with expanded G4R2 can also be translated through the repeat-associated non-ATG (RAN) translation mechanism to synthesize dipeptide (Gly-Ala) repeat proteins that are liable to form toxic aggregates in neurons [213,214,215].

Actually, many RNA molecules with repeated expansion disorders can form toxic RNA aggregates regardless of their locations in exons, introns, or untranslated regions (UTRs) [216]. These RNA with noncanonical structures, such as G-quadruplexes, can stack onto each other to form insoluble RNA foci in the nuclei of cells and tissues, which increase nuclear stress [217], sequester RPBs [218], impair nucleocytoplasmic transport in neuronal cells [219], and ultimately lead to neurodegenerative disorders. Jain et al. demonstrated that repeated RNA expansions with a similar critical repeat number to neurological diseases, such as G4C2 in the *C9ORF72*, could serve as templates for multivalent base-pairing and subsequently promote an RNA solid-to-gel transition in vitro. Agents that disrupted this RNA gelation could also dissolve the foci in human cells formed by the RNA with expanded repeats, suggesting that sequence-specific RNA gelation could also contribute to neurodegenerative diseases [220].

TDP-43 can also bind G-quadruplex structure in mRNAs and subsequently deliver them to specific subcellular compartments in neuronal cells for local translation. The M337V mutation at the C-terminal Gly-rich domain of TDP-43 abolishes its G-quadruplex binding affinity and subsequently deprives its ability to transport G4-containing mRNAs neurites, which is an etiological cause of ALS and FTD [221].

FUS is also a protein bearing various cellular functions including transcriptional regulation, RNA splicing, mRNA transport and translation, and DNA repair [222]. Meanwhile, FUS regulates mRNA nonsense-mediated decay (NMD), a cellular mechanism to quickly degrade defective RNAs with premature stop codons; thus, mutations in FUS can dampen protein synthesis efficiency to trigger hyperactive NMD, leading to aberrant cellular protein homeostasis, which may induce the formation of protein inclusions to sequester regulatory proteins [223]. Ishiguro et al. reported that RNA G-quadruplexes could promote the liquid-to-solid transition of FUS leading to FUS aggregation. Consistently, among various FUS variants, the *FUS P525L* mutant discovered in juvenile ALS patients manifested the highest binding affinity to RNA G-quadruplexes, which highlighted the contributions of RNA secondary structures and dysfunctional RPBs to ALS pathogenesis [224]. In line with this study, Wang et al. recently demonstrated that RNA G-quadruplexes could promote liquid-to-solid phase transition of short RNAs, which could promote neuronal disorders [225].

The X-linked Fragile X Mental Retardation 1 (*FMR1*) gene can also be abnormally expanded to over 200 CGG triplet repeats (versus about 30 repeats in normal individuals) in its 5′-UTR, which is almost the exclusive pathological cause of Fragile X Syndrome (FXS), a neurodevelopmental disease characterized by intellectual disability, hyperactivity and anxiety of the affected individuals. The highly repeated CGG sequence in the FMR1 genomic locus leads to increased DNA methylation, transcriptional inactivation and loss of its gene product FMRP [226]. FMRP harbors three RNA binding domains, including two KH domains and one RGG domain [227]. FRMP represses mRNA translation through binding to the G-quadruplexes in target mRNAs [228]. FMRP deficient neurons exhibited retarded maturation and abnormal morphology of the dendritic spines [229].

Overall, aberrant protein phase separation, which leads to the loss of protein function and the formation of toxic aggregates, is the common phenomenon of different neurodegenerative disorders and contributes to disease progression. Pathologically, the aggregation of different amyloidogenic proteins can be triggered by various and distinct reasons, including mutations, expression levels, PTMs, aberrant subcellular localizations, altered binding to nucleic acids, and environmental changes, among others.

## 4. Techniques to Study Protein Phase Separation and Aggregation

Cellular membraneless compartments formed by phase separation mechanism have been described using different nomenclatures, including nuclear or cytoplasmic bodies, condensates, granules, speckles, puncta, aggregates, and inclusions [230]. Various techniques have been used to detect phase-separated condensates in vitro and in a cellular environment (so-called “in cellulo” in recent years). Among them, microscopy is a common technique to directly visualize biomolecules in their phase-separated or aggregated status and can be used to primarily discriminate them. Typically, phase-separated condensates of normal biomolecules look like liquid droplets or well-shaped spheres in vitro, while the outlines of cellular puncta may be irregular. With the changes of samples expressing or silencing a specific gene, carrying a mutation, or staying under different environmental conditions, such as pH, temperature, incubation period, or the presence or absence of crowding or dissolving agents, the droplets may alter their shape, size, or number. Bright-field microscopes can be readily utilized for the observation, but fluorescence microscopes can better display molecular condensation if examined molecules are conjugated by fluorescent tags, which will be especially helpful for simultaneously detecting the co-condensation of two or more molecules. For the determination of molecular condensation in cellulo, it requires the use of fluorescent probes or secondary antibodies associated with specific primary antibodies, or fusion with fluorescent proteins, such as mCherry and green fluorescent proteins (mCherry and EGFP, respectively). Fluorescence recovery after a photobleaching (FRAP) assay is frequently conducted to evaluate the liquidity or molecular dynamics of the condensates. Generally, normal biomolecular condensates manifest fast fluorescence recovery or high molecular dynamics, but aged condensates or aberrant aggregates with solid- or gel-like states exhibit retarded FRAP due to slow internal molecular mobilities [231].

More advanced technologies have been applied to the structural analysis of biomolecular condensation and aggregation. Magnetic resonance imaging (MRI) is an imaging approach that employs radiology with strong magnetic fields and gradients, and radio waves to generate images of organs and detect physiological processes in live bodies. The structures of many amyloidogenic protein aggregates, such as α-syn, Aβ, tau, FUS, TDP-43, and C9ORF72, have been analyzed at an atomic resolution using the MRI approach [232,233,234,235,236,237].

Notably, advances in recent years have demonstrated the feasibility of using cryo-electron microscopy (cryo-EM) to resolve the aggregate structures of many amyloidogenic proteins at a near-atomic resolution in tissues and organs excised from living organisms, which are termed as ex vivo studies [238]. For example, the cryo-EM structures of tau filaments from AD individuals indicated that the paired helical and straight tau filaments, which account for neurofibrillary lesions, had the same protofilaments but their interprotofilament packing was different [239]. Schweighauser et al. also employed cryo-EM approach to reveal that two types of filaments consisting of two different protofilaments were present in the brains of patients with multiple system atrophy. α-syn filaments from these patients showed a distinct structure from those bearing dementia with Lewy bodies [240]. Furthermore, other research groups have used cryo-EM to successfully analyze the structures of Aβ and TDP-43 aggregates [241,242].

To date, the understanding of biomolecular condensation and aggregation associated with neurodegenerative diseases is by no means complete, and there is an urgent need for new techniques and strategies to be invented and developed for researcher to gain more insightful knowledge, especially at ex vivo and in vivo levels.

## 5. Treatments of Neurodegenerative Diseases by Reducing Protein Aggregation

Aberrant phase separation promotes protein aggregation that is a hallmark of neurodegenerative disorders. Various approaches have been explored to modulate protein phase separation and aggregation with a goal of seeking for therapeutic candidates and strategies of neurodegenerative diseases.

### 5.1. Targeting the PTMs of Amyloidogenic Proteins

PTMs of key residues may alter protein interactions that are important for the formation of fibrillar inclusions in neuronal cells. Among these PTMs, the phosphorylation of Aβ, α-syn, and TDP-43 plays a critical role in the pathogenesis of various neurodegenerative diseases, such as AD, PD, and ALS. Strategies targeting the phosphorylation enzymes and pathways of these proteins have been extensively explored to prevent and reduce the accumulation and aggregation of these proteins [243]. Phosphorylation is intricately involved in ALS progression. While phosphorylation prevents the formation of SOD1 toxic aggregates, it increases TDP-43 oligomerization and fibrillization in neuronal cells of ALS patients. With this dilemma, selectively targeting kinases is necessary to ameliorate ALS by reducing the phosphorylation of a specific protein without triggering the aggregation of others. After analyzing a number of kinases, Ko et al. discovered that CK1δ and CK1ε interacted with TDP-43 and played critical roles in promoting TDP-43 hyperphosphorylation at Ser409 and Ser410, leading to its aberrant aggregation [244]. In an ALS mouse model using the transgene of the TDP-43-A315T, treatment of IGS-2.7, a brain penetrant inhibitor of CK-1δ, could reduce TDP-43 phosphorylation and preserve motor neurons in the anterior horn at the lumbar level. In isolated cells derived from ALS patients, the treatment could also decrease aberrant TDP-43 aggregation by restoring the normal states of its phosphorylation and subcellular localization [245].

Many studies have demonstrated the molecular tweezer CLR01 for its activity in eliminating aggregation of amyloidogenic proteins. Molecular tweezers are synthetic molecular receptors. Each molecular tweezer has an open cavity with two interaction sites for substrate binding and a flexible spacer [246]. Sinha et al. reported that CLR01 could specifically bind Lys residues of Aβ and disrupt the hydrophobic and electrostatic interactions of its residues, which are required for the nucleation, oligomerization, and fibrilization. CLR01 interacts with Aβ at its monomer stage and regulates its assembly process to form nontoxic structures instead of amyloid fibers [247]. CLR01 could also remodel Aβ42 oligomerization by compacting its dimer and tetramer structures, which consequently decreased higher-order oligomers [248]. In a tau transgenic mouse model, CLR01 could reduce tau pathology and improve defective behaviors in P301S-tau transgenic mice, suggesting its attractive candidacy in AD treatment [249]. Additionally, CLR01 exhibited protective effects on dopaminergic neurons in a PD mouse model [250]. In a study by Vopel et al., CLR01 bound the polyQ tract produced by *HTT*-exon1 and subsequently modulated its structural rearrangement that was unfavorable for aggregation, suggesting its therapeutic potential in the treatment of HD [251]. Additionally, CLR01 significantly decreased SOD1 misfolding in the spinal cord of ALS mice, likely through binding to the lysines 61 and 92 [252,253].

### 5.2. Targeting the Genes Encoding Amyloidogenic Proteins

Targeting key genes or proteins involved in protein aggregation is also a promising exploratory direction for the treatment of neurodegenerative diseases. Ekman et al. employed the CRISPR-Cas9 approach to disrupt the mutant *HTT* gene, which could reduce about 50% of neuronal inclusions and markedly improve the survival and motor deficits in a HTT mouse model [254]. *Ataxin 2* (*ATXN2*) is a gene encoding a polyQ protein that associates with TDP-43 and regulates its cytoplasmic mislocalization in the spinal cord neurons of ALS patients [255]. In an ALS mouse model, Becker et al. used antisense oligonucleotides (ASOs) to target *ATXN2* expression and subsequently reduced TDP-43 aggregation. Phenotypically, *ATXN2* knockdown could significantly extend the survival and improve the motor function of ALS mice [256].

RBP mutations contribute to the onset and progression of various neurodegenerative disorders; thus, targeting RBPs or their RNA partners has become plausible strategies in the treatment of these diseases, and its progresses have been very encouraging in recent years [257]. Hua et al. designed a set of ASOs to specifically block an *SMN2* intronic splicing silencer element and one of them robustly increased exon 7 inclusion during its pre-mRNA splicing. Strikingly, a single intracerebroventricular injection of the ASO for the embryonic or neonatal SMA mice could rescue the tail and ear necrosis and significantly improve the survival and neuromuscular function [258,259]. Importantly, as a derivative of this ASO, Nursinen has been used in multiple clinical trials and has exhibited beneficial effects on SMA patients [260,261,262,263]. Taking advantage of CRISPR technology, Liu et al. used a dead-Cas9 to bind the repeat expansion of the *FMR1* and subsequently promoted its demethylation and gene reactivation, which rescued electrophysiological abnormalities and restored the normal phenotype in a FXS mouse model [264]. CRISPR/Cas9 was also employed to delete the expanded CGG-repeat to reactivate FMR1 [265]. A number of other approaches have also been developed to correct aberrant RNA metabolism or intervene disordered RBPs, with promising potentials to be applied in the therapies of neurodegenerative diseases [257].

### 5.3. Other Emerging Therapeutic Approaches

The onset of neurodegenerative diseases can be caused by multiple and concurrent factors through numerous mechanisms, which impedes the development of effective therapeutic strategies. Early studies indicated a 0.4% success rate for the clinical trials of AD therapies [266]. Nevertheless, many new approaches and strategies have emerged to manifest their therapeutic potentials for different neurodegenerative diseases.

As a relatively non-invasive treatment, stem cell therapy uses the cells or products of live stem cells to replace or stimulate the proliferation and differentiation of damaged or defective cells, which may alleviate the neurodegenerative symptoms. Yoo et al. transplanted hematopoietic stem cells into mice, which could effectively replace defective microglia in AD mice and restore microglial function [267]. Another report by Mishra et al. also demonstrated that systemic WT hematopoietic stem and progenitor cell transplantation into AD mice could diminish microglia activation, decrease Aβ plaque quantity in the hippocampus, reduce cortex neuroinflammation, and prevent memory loss and neurocognitive impairment [268]. Additionally, exploration of stem cell therapies for PD has also been conducted by different research groups [269].

Since neurodegenerative diseases are caused by protein disorders, targeting these proteins to promote their degradation is a plausible tactic to treat the diseases. Proteolysis targeting chimera (PROTAC) is an approach to design and create a molecule that can specifically degrade and remove a protein from cells. To date, the PROTAC approach has been used to successfully target many neurodegenerative disease-relevant proteins, including α-syn, tau, mHTT, TDP-43, and GSK-3, among others [270]. For example, Tseng et al. designed multiple PROTACs to target the C-terminal of TDP-43 (C-TDP-43). One of these PROTACs could decrease the aggregates and reduce the cytotoxicity caused by C-TDP-43 through promoting its ubiquitination and proteolytic degradation [271]. In another report, Zhu et al. also discovered an effective dual PROTAC degrader that could eliminate α-syn aggregates and total tau simultaneously through the ubiquitin-proteasome system. In a PD mouse model, the PROTAC degrader could reduce the levels of aggregated protein and protect the dopaminergic neurons from cell injury [272]. In a recent study, Miller et al. designed a therapeutic strategy “‘RING-Bait’’ that employed an aggregating protein sequence fused with an E3 ubiquitin ligase. The RING-Bait based on the tau sequence was recruited into aggregates, where the dimerized RING domains could activate its E3 activity to specifically degrade tau aggregates but spare soluble tau, leading to reduced tau pathology and improved motor function [273].

Immunotherapy has become an attractive approach at the prime position of therapeutic development for neurodegenerative diseases. Generally, immunotherapies employ the immune system of patients to treat the diseased conditions using vaccines or monoclonal antibodies, which are classified as active immunization (stimulating the immune system to produce endogenous antibodies against the target proteins) and passive immunization (directly administering antibodies against the targets), respectively. AN1792 is the first vaccine for AD immunotherapy, containing synthetic full-length Aβ peptide with the saponin QS-21 as an adjuvant. In *APP* transgenic mice, AN1792 could reduce Aβ plaque burden and preserve the cognitive function [274,275]. Recently, Hou et al. created a monoclonal antibody against LILRB4, an inhibitory receptor of the immunoglobulin (Ig) superfamily, which was highly expressed in the microglia and covered Aβ plaques of AD patients. The administration of this antibody in an AD mouse model could reduce Aβ levels, mitigate some abnormal behaviors caused by Aβ aggregation, enhance microglia activity, and downregulate the expression of interferon-induced genes [276]. Furthermore, a number of studies for PD immunotherapy have been conducted, which have aimed to decrease microglial activation, suppress inflammasome, adjust immunosuppression, or target specific receptors [277].

Overall, the treatment landscape for neurodegenerative diseases encounters various challenges. Primarily, conditions of AD and PD lack definitive cures but rely mainly on pharmaceuticals and rehabilitative treatments to relieve the symptoms and slow down the progression of neurodegenerative diseases. In addition, many treatment strategies only ameliorate the disease symptoms and improve life quality of the patients but fall short in intervening the fundamental causes of disease progression. In this context, the integration of knowledge and technology related to protein phase separation holds great promise to address these challenges.

## 6. Conclusions and Future Directions

Neurodegenerative diseases impose immense and devastating impacts on the life quality of patients and their families. In the past decades, understanding of the disease mechanisms has been significantly advanced in basic research and preclinical exploration, due to unremitting endeavors of researchers and tremendous investments of many governments. However, large gaps still exist in portraying the exhaustive landscapes for the onset, development, and aggravation of most neurodegenerative diseases. In the past decade, exquisite detection, molecular simulation, and structural analysis of protein monomers and oligomers have greatly improved our perception and understanding for the etiological protein aggregation and pathological symptom progression of the patients. Especially, protein aggregation and phase separation are recognized as interrelated cellular processes linked to protein phase transitions and subsequent neurodegenerative disorders. Noteworthily, the analysis of primary sequences indicates that several amyloidogenic proteins do not contain significant IDRs (Figure 2), suggesting that disordered motifs are not the major cause of phase separation or aggregation for all proteins. We recently reported that zinc fingers also contribute to protein condensation [278,279]. Ample evidence indicates that aberrant protein aggregation proceeds through their phase separation. Each neurodegenerative disease may have its own representative aggregation-prone proteins whose aggregation is the major pathological causes of the disease. Meanwhile, some proteins, such as Aβ, tau, and α-syn, may be involved in different neuronal disorders. Mutations, aberrant PTMs, altered binding patterners, elevated expression, and mislocalization are representative causes for the formation of protein inclusions associated with the development of these diseases.

Many potential directions and unanswered questions in the research and treatments of neurodegenerative diseases deserve future exploration. First, we still have limited understanding of the diverse and complicated mechanisms underlying the onset and progression of these diseases. Related to the topic of this review, future research should continue to explore the relationship of protein phase separation and aggregation with neurodegenerative diseases to better delineate the development and progression of these diseases. Especially, the transition from phase separation to protein aggregation is a complicated and progressive process that can be modulated and intervened by different factors. Understanding of this process can provide new insights in formulating more effective prevention and personalized treatment strategies.

Second, early and precise diagnoses are ideal and prime for the prevention and effective intervention of neurodegenerative diseases. Currently, the majority of tested agents in clinical trials showed promising efficacy to the neurodegenerative diseases at the early development but were ineffective to the diseases at their late stages. Thus, early diagnosis is crucial to identify the susceptible individuals before the commencement or at the early stages of the diseases [280]. However, unlike many other human diseases, the lesions of neurodegenerative disorders, such as AD and PD, are located in the brain, which is difficult or unavailable for sample collection, unless during the autopsy after patients’ demise. Most aforementioned aggregation-prone proteins are bona fide biomarkers of corresponding neurodegenerative diseases. In addition, other indicators or symptoms, including unbalanced proteostasis, dysregulated synaptic and neuronal networks, abnormal cytoskeletal structures, aberrant energy metabolism, inflammation, and DNA and RNA defective changes, among many others, have also been considered as hallmarks of neurodegenerative diseases [4]. These hallmarks or biomarkers are genuine subjects for the development of novel strategies for early and accurate diagnosis of different neurodegenerative diseases.

Third, the development of suitable disease models is pressingly needed for mechanistic understanding and therapeutic discovery of neurodegenerative diseases. Preclinical studies generally employ rodent models, mostly mice and rats. However, the development and function of rodent brains are different from human brains. Thus, this may bring difficulties to interpret the behavioral deficits, especially altered cognitive, emotional, and language behaviors. Additionally, due to the physiological difference, animal models cannot precisely manifest the full complexity of human diseases. The generation of animal models typically utilize genetic manipulations, including gene overexpression, knockout, or genomic DNA mutagenesis; however, many neurodegenerative diseases are sporadic without any familial or genetic clue. Additionally, neurodegenerative diseases are age-related disorders; thus, the short lifespans of rodents make them impossible to manifest the process of spontaneous disease onset or fully develop pathological hallmarks.

Noteworthily, researchers have developed the brain organoids using human-induced pluripotent stem cells that can serve as a model to mimic the three-dimensional structure and recapitulate the major developmental stages of the human brain. Obviously, this organoid model faces crucial limitations compared to the rodents, but it possesses superior advantages in drug screening to quickly identify novel candidate targets for further evaluation in neurodegenerative disease therapies [281].

Overall, despite the significant progresses in the research and therapies of neurodegenerative diseases, we are still facing many fertile topics to explore and enormous unanswered questions.

## Figures and Tables

**Table 1:** Common neurodegenerative diseases and associated key regulatory proteins.

Diseases	Key Proteins	Genes	Functions	Consequence of Mutations	References
AD	Aβ (or APP)	*APP*	Cell surface receptor	Increase of Aβ, especially the Aβ-42 peptide	[23]
	γ-secretase	*PSEN1/* *PSEN2*	APP cleavage to form Aβ	Increased ratio of Aβ-42/Aβ-40 to form amyloid plaques	[24]
	tau	*MAPT*	A microtubule- associated protein	Formation of tau aggregates toxic to neuronal cells	[25]
PD	α-syn	*SNCA*	A neuronal protein for synaptic vesicle trafficking and neurotransmitter release	Formation of insoluble amyloid fibrils in Lewy bodies	[26]
	GBA1	*GBA1*	Glucocerebrosidase β1	Accumulation of glycolipid substrates	[27]
	ferritin	*FTH*, *FTL*	Conversion of Fe^2+^ to Fe^3+^ iron	Generation of hydroxyl radicals for PD pathogenesis	[28,29]
HD	HTT	*HTT*	Cellular signaling and axonal transport	Formation of aggregates to generate Huntington’s bodies	[30,31]
ALS	SOD1	*SOD1*	Destruction of free superoxide radicals	Aggregation leading to ALS development	[32]
	TDP-43	*TDP-43*	A transcriptional factor	Altered subcellular localization to form aggregates	[33,34]
	FUS	*FUS*	Transcription, RNA processing, DNA repair	Altered subcellular localization to form cytoplasmic inclusions	[35]
AMD	ApoE	*APOE*	A lipoprotein as a cholesterol transporter	ApoE polymorphism isoforms with different risks for AMD development	[36,37]
	RPE65	*RPE65*	A retinoid isomerohydrolase	Increase of RPE65 aggregation propensity	[38,39]

## Data Availability

Data are contained within the article and cited references.
